# Axial O Atom‐Modulated Fe(III)‐N_4_ Sites for Enhanced Cascade Catalytic ^1^O_2_‐Induced Tumor Therapy

**DOI:** 10.1002/advs.202307254

**Published:** 2024-07-01

**Authors:** Hongji Liu, Biao Yu, Pengqi Yang, Yang Yang, Zhiming Deng, Xin Zhang, Kai Wang, Hui Wang

**Affiliations:** ^1^ High Magnetic Field Laboratory Hefei Institutes of Physical Science Chinese Academy of Sciences Hefei Anhui 230031 P. R. China; ^2^ State Key Laboratory of Chemo/Biosensing and Chemometrics College of Chemistry and Chemical Engineering Hunan University Changsha Hunan 410082 P. R. China; ^3^ Department of Obstetrics and Gynecology The First Affiliated Hospital of Anhui Medical University Hefei Anhui 230022 China; ^4^ NHC Key Laboratory of Study on Abnormal Gametes and Reproductive Tract Anhui Medical University Hefei Anhui 230022 China; ^5^ Hefei National Research Center for Physical Sciences at the Microscale and Department of Materials Science and Engineering University of Science and Technology of China Hefei 230026 China; ^6^ State Key Laboratory of Pharmaceutical Biotechnology School of Life Sciences Nanjing University Nanjing Jiangsu 210023 P. R. China

**Keywords:** axial O coordination, cascade catalytic therapy, ferroptosis, iron single atom nanozymes, singlet oxygen

## Abstract

The rational construction of efficient hypoxia‐tolerant nanocatalysts capable of generating singlet oxygen (^1^O_2_) without external stimuli is of great importance for tumor therapy. Herein, uniformly dispersed and favorable biosafety profile graphitic carbon nitride quantum dots immobilized with Fe‐N_4_ moieties modulated by axial O atom (denoted as O‐Fe‐N_4_) are developed for converting H_2_O_2_ into ^1^O_2_ via Russell reaction, without introducing external energy. Notably, O‐Fe‐N_4_ performs two interconnected catalytic properties: glutathione oxidase‐mimic activity to provide substrate for subsequent ^1^O_2_ generation, avoiding the blunting anticancer efficacy by glutathione. The O‐Fe‐N_4_ catalyst demonstrates a specific activity of 79.58 U mg^−1^ at pH 6.2, outperforming the most reported Fe‐N_4_ catalysts. Density functional theory calculations demonstrate that the axial O atom can effectively modulate the relative position and electron affinity between Fe and N, lowering the activation energy, strengthening the selectivity, and thus facilitating the Russell‐type reaction. The gratifying enzymatic activity stemming from the well‐defined Fe‐N/O structure can inhibit tumor proliferation by efficiently downregulating glutathione peroxidase 4 activity and inducing lipid peroxidation. Altogether, the O‐Fe‐N_4_ catalyst not only represents an efficient platform for self‐cascaded catalysis to address the limitations of ^1^O_2_‐involved cancer treatment but also provides a paradigm to enhance the performance of the Fe‐N_4_ catalyst.

## Graphical abstract

The introduction of an axial O atom can adjust the relative position and electron affinity between Fe and N atoms, which causes d‐band center of Fe‐N_4_ moiety to shift toward Fermi level, thus enhancing the electrons attraction, reducing the energy barriers, strengthening the selectively and catalytic efficiency of Russell reaction, resulting in a tumor inhibition rate as high as 86.10%.

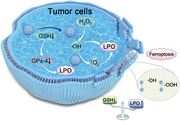

## 1 Introduction

Singlet oxygen (^1^O_2_)‐elevated strategy is undoubtedly a favorable modality for inhibiting the proliferation of malignant tumors due to its long lifetime (10^−6^ – 10^−3^ s) and selectivity to electron‐rich biomolecules.^[^ ^1^ ^]^ However, phototoxicity and low effective external stimulation have made the clinical practice of photodynamic therapy (PDT, the most widespread considered ^1^O_2_‐mediated tumor therapy) an almost insurmountable task.^[^ ^2^ ^]^ With the development of nanotechnology, non‐photo‐induced ^1^O_2_‐related therapeutic approaches, including sonodynamic therapy,^[^ ^3^ ^]^ microwavedynamic therapy,^[^ ^4^ ^]^ and radiodynamic therapy,^[^ ^5^ ^]^ are used to overcome the inherent limitations of PDT. Nevertheless, together with PDT, the broad applications of non‐photo‐induced ^1^O_2_‐producing therapeutic strategies may be hampered by the hypoxia in solid tumors and the complexity of tumor microenvironment (TME).^[^ ^6^ ^]^ As a potential alternative to external stimuli tactics, Russell‐type chemodynamic therapy (CDT) offers an oxygen‐independent approach to sensitize ^1^O_2_ generation, circumventing the normal tissue damage associated with exogenous stimulation.^[^ ^7^ ^]^ Up to date, only Cu‐based and Mo‐based nanomaterials have been reported to be used in the Russell‐type CDT,^[^ ^8^ ^]^ while other metals or metal‐free nanozymes afford Russell reaction inertness. Therefore, the development of a new Russell‐type CDT reagent is valuable to enhance the practical feasibility of the ^1^O_2_‐generating tumor therapy.

Single‐atom enzymes (SAEs) inspired by natural enzymes have increasing potential in biomedical applications, such as biosensing and cancer therapy.^[^ ^9^ ^]^ More importantly, SAEs provide an opportunity for the design of Russell‐type nano reagents due to the tunable electronic/geometric structure, uniform active sites, and alternative energy pathways.^[^ ^10^ ^]^ However, the symmetric electron distribution of typical SAEs with Metal‐N_4_ configurations makes the catalytic performance unsatisfactory for high therapeutic efficiency.^[^ ^11^ ^]^ Oriented by the five‐coordinated structure of horseradish peroxidase, introducing axial heteroatom (N or O) coordination in Metal‐N_4_ configuration may offer an innovative strategy to improve anti‐cancer efficiency because of their improved electronic property, catalytic activity, selectivity, and durability.^[^ ^12^ ^]^ Moreover, another important factor affecting anti‐cancer efficiency is the overexpressed glutathione (GSH) in TME.^[^ ^13^ ^]^ In parallel, the high‐valence metal ions, such as Cu(II) or Fe(III), preferentially react with GSH and continuously supply low‐valence metal for the following reaction.^[^ ^14^ ^]^ Enlightened by the above analysis, the reasonable design of axial O atom modulated Fe(III)‐N_4_ SAEs is expected to achieve a highly efficient inhibition rate in the ^1^O_2_‐generating tumor therapy.

**Figure 1:**
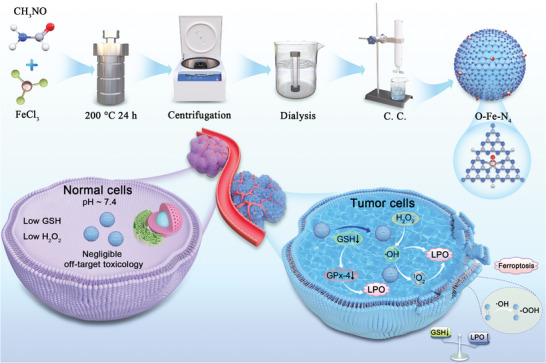
Synthetic strategy of O‐Fe‐N_4_ SAEs for enhanced cascade catalytic ^1^O_2_‐induced therapy.

Herein, we show a kind of Fe‐based SAEs anchored on graphitic carbon nitride quantum dots (CNQDs) with axial O atom engineered Fe(III)‐N_4_ moieties (denoted as O‐Fe‐N_4_) for realizing efficient H_2_O_2_ Russell reaction to ^1^O_2_ at hypoxic environment without external stimulus (**Figure** **1**). The well‐defined five‐coordinated structure in the O‐Fe‐N_4_ catalyst provides an opportunity to achieve unambiguously catalytic activity. Outstandingly, the O‐Fe‐N_4_ nanozyme is identified to hold self‐cascade enzymatic performance: glutathione oxidase (GSHOD) mimicking activity (specific activity, SA = 2.15 U mg^–1^) and reactive oxygen species (ROS)‐induced performance (SA = 79.58 U mg^–1^), avoiding the loss of ROS. Density functional theory (DFT) calculations elucidate that the introduction of an axial O atom causes the d‐band center of the Fe‐N_4_ site to shift toward the Fermi level, reducing the reaction activation energy, leading to higher selectivity and production efficiency of ^1^O_2_. In vivo and in vitro experiments show that glutathione peroxidase 4 (GPx‐4) reduction caused by GSHOD activity and lipid peroxidation (LPO) mediated by ROS production jointly inhibited triple‐negative breast cancer cell proliferation. Furthermore, the O‐Fe‐N_4_ catalyst demonstrates a new paradigm to catalytic generation ^1^O_2_ for tumor treatment without external stimulus.

## 2 Results and Discussion

### 2.1 Synthesis and Characterization of O‐Fe‐N_4_

**Figure 2:**
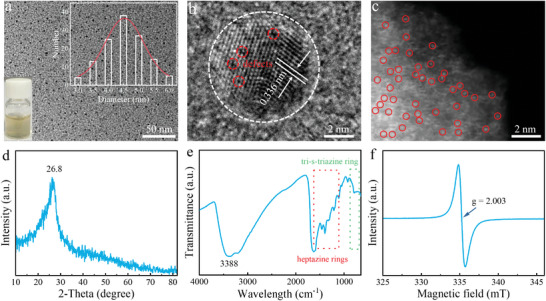
Morphology and characterization of O‐Fe‐N_4_. a) TEM image, size distribution (inset), and optical photo (inset) of O‐Fe‐N_4_. b) HR‐TEM image of an O‐Fe‐N_4_ nanoparticle, the red dotted circles represent the locations of vacancy defects. c) AC‐STEM image of O‐Fe‐N_4_, with some of the Fe atoms highlighted by the red circles. d–f) XRD patternc(d), the FT‐IR spectrum (e), and the EPR spectrum (f) of O‐Fe‐N_4_, respectively.

The O‐Fe‐N_4_ enzyme was prepared by pyrolyzing and carbonizing the Fe(II)‐formamide dispersion. In brief, the amine groups and carbonyl groups in formamide molecules could be bonded to each other through nucleophilic addition to form chain macromolecules,^[^ ^15^ ^]^ and the presence of N atoms in macromolecules was used to chelate iron cations.^[^ ^16^ ^]^ Finally, the O‐Fe‐N_4_ catalyst was obtained under continuous carbonization in a high‐pressure reactor. The outer contour of O‐Fe‐N_4_ was a spherical configuration with a monodisperse of 4.5‐nm‐sized nanoparticles (**Figure** **2**a). The lattice fringe was measured to be 0.336 nm (Figure 2b), corresponding to bulk g‐C_3_N_4_,^[^ ^17^ ^]^ which confirmed that the matrix of O‐Fe‐N_4_ was CNQDs. Moreover, obvious defects could be observed in the high‐resolution (HR) transmission electron microscopy (TEM) image, which were conducive to the loading of Fe atoms. Immediately after, Fe atoms in CNQDs were identified by aberration‐corrected scanning transmission electron microscopy (AC‐STEM, Figure 2c). Results revealed clearly that Fe atoms (small bright dots, <0.3 nm, labeled by red circles) were randomly dispersed on the CNQDs substrate. The successful preparation of CNQDs matrix was further confirmed by the intense peak evident in the X‐ray diffraction (XRD) pattern (Figure 2d).^[^ ^18^ ^]^ The C‐N‐C stretching in a specific region (1100–1600 cm^−1^) together with the fingerprint peak of the triazine ring (790 cm^−1^) in the Fourier transform infrared (FT‐IR) spectrum further indicated the successful synthesis of CNQDs substrate (Figure 2e).^[^ ^19^ ^]^ Importantly, the iron‐free absorption peaks in the XRD pattern and FT‐IR spectrum preliminarily proved the successful preparation of SAEs. In addition, the unsaturated C and N vacancies (Figure 2f; Figure S1, Supporting Information) of O‐Fe‐N_4_ provide the structural basis to anchor Fe species.^[^ ^20^ ^]^

### 2.2 Active Site Structure Analysis of O‐Fe‐N_4_

**Figure 3:**
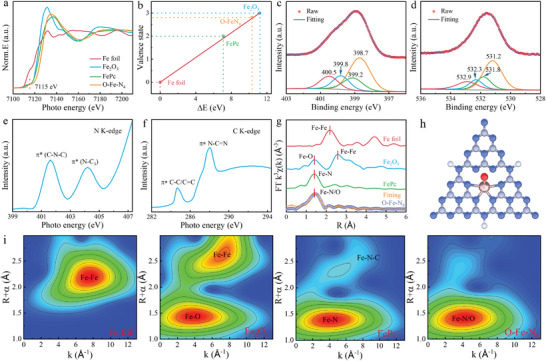
Active site structure analysis of O‐Fe‐N_4_. a) XANES spectra at the Fe K‐edge of O‐Fe‐N_4_, Fe‐Pc, Fe_2_O_3,_ and Fe foil, respectively. b) The average oxidation states of Fe in O‐Fe‐N_4_ from XANES spectra. c,d) XPS high‐resolution survey scans of N1s (c) and O1s (d) in O‐Fe‐N_4_. e,f) N K‐edge XANES spectrum (e) and C K‐edge XANES spectrum (f) of O‐Fe‐N_4_. g) k^3^‐weighted Fourier transforms of O‐Fe‐N_4_, Fe‐Pc, Fe_2_O_3_ and Fe foil, respectively. The scatter is the FT‐EXAFS fitting curve of O‐Fe‐N_4_. h) Atomic structure model of O‐Fe‐N_4_ catalyst. i) WT of O‐Fe‐N_4_ in comparison with FePc, Fe_2_O_3_, and Fe foil samples, respectively.

The Fe content in O‐Fe‐N_4_ SAEs could be measured and calculated to be 5.26 wt.% via inductively coupled plasma atomic emission spectrometry (ICP‐AES). The X‐ray photoelectron spectroscopy (XPS) survey showed that iron species existed on the surface of O‐Fe‐N_4_ with a proportion of ≈1 at.% (Figure S2, Supporting Information), which was consistent with the ICP‐AES result. The pre‐edge peak (7115 eV) stemming from the 1s→3d transition,^[^ ^21^ ^]^ and the absorption edge energy in K‐edge X‐ray absorption near‐edge structure (XANES, **Figure** **3**a) suggested the electronic structure of Fe species (δ close to +3, Figure 3b) in O‐Fe‐N_4_,^[^ ^22^ ^]^ which was similar to that of XPS results (Figure S3, Supporting Information) and XANES linear combination fitting analysis (Figure S4 and Table S1, Supporting Information).^[^ ^23^ ^]^ Moreover, the pre‐edge peak also implies that FeN_4_ moieties exist in O‐Fe‐N_4_ due to the similarity to FePc. Both the Fe‐N fitting peak in the N1s spectrum (Figure 3c) and the Fe‐O fitting peak in the O1s spectrum (Figure 3d) further illustrated that the Fe species could be anchored in the CNQDs matrix via N or O atoms.^[^ ^24^ ^]^ Soft XANEs were then conducted to clarify the electronic structure of O‐Fe‐N_4_. The π* characteristic resonance of 401.6 eV in the N K‐edge spectrum (Figure 3e) was associated with the aromatic C‐N‐C of tri‐s‐triazine heteroring, while the other π* characteristic feature was attributed to N‐(C)_3_.^[^ ^25^ ^]^ Both C K‐edge spectrum (Figure 3f) and C 1s XPS spectrum (Figure S5, Supporting Information) co‐pointed to the π* C═C/C─C transitions and π* N─C═C bond in O‐Fe‐N_4_.^[^ ^26^ ^]^ As illustrated in the Fourier transform extended X‐ray absorption fine structure (EXAFS) spectrum (Figure 3g), the primary peak of O‐Fe‐N_4_ at around 1.41 Å could be observed, which agreed with the first coordination sphere of Fe‐N in FePc or Fe‐O coordination environment in Fe_2_O_3_. Notably, the obvious feature of Fe‐Fe bonds (2.18 Å or 2.56 Å) was not detected, implying the Fe species were atomically dispersed in the CNQDs matrix. The quantitative EXAFS fitting curves (Figures S6–S9 and Table S2, Supporting Information) demonstrated the isolated Fe species in the form of O_1_‐Fe‐N_4_ moiety in O‐Fe‐N_4_, with the average Fe‐N bond length and Fe‐O bond length of 1.98 Å and 2.01 Å, respectively (Figure 3h). The atomically O‐Fe‐N_4_ was further confirmed by higher resolution Morlet wavelet transformation (Figure 3i), in which only one intensity maxima at 4.1 Å^–1^ originated from Fe‐O/N scattering path. The above procedures indicated that Fe species existed in the form of five coordinated single atoms in CNQDs matrix, i.e., 4 N atoms into tri‐s‐triazine and 1 O atom located out the planar.

### 2.3 Self‐Cascade Enzymatic Performance of O‐Fe‐N_4_

**Figure 4:**
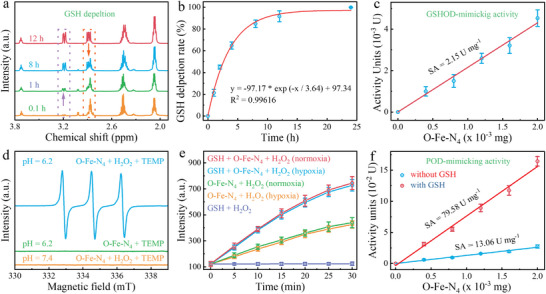
Catalytic properties of O‐Fe‐N_4_. a) ^1^H NMR spectra of GSH and GSSG in reaction progress between GSH and O‐Fe‐N_4_. b) The GSH depletion in the presence of O‐Fe‐N_4_ within 24 h (*n* = 3). c) The SA of O‐Fe‐N_4_, with GSH as a substrate (*n* = 3). d) EPR spectra of ^1^O_2_ in different systems with TEMP as the trapping agent. e) Time‐dependent ^1^O_2_ generation under normoxic and hypoxic conditions detected by SOSG (*n* = 3). f) Comparison of the SA of O‐Fe‐N_4_ with or without GSH (*n* = 3). Values are expressed as mean ± standard deviation.

The valance state (δ close to +3) of Fe species endowed O‐Fe‐N_4_ superior cascade enzymatic performance, including GSHOD‐like and subsequent peroxidase (POD)‐like activity. As revealed in **Figure** **4**a, the proton nuclear magnetic resonance (^1^H NMR) spectra showed that the GSH‐related peaks gradually weakened over time, while the corresponding feature of glutathione disulfide (GSSG) gradually enhanced. Besides, the GSH depletion behavior of O‐Fe‐N_4_ could also be verified via the Ellman reagent of which O‐Fe‐N_4_ completely converted GSH to GSSG within 24 h (Figure 4b), indicating the great potential of O‐Fe‐N_4_ as a GSHOD mimic. Moreover, the SA value for GSHOD‐mimicking performance of O‐Fe‐N_4_ was determined to be 2.15 U mg^−1^ (Figure 4c). Downstream products (Fe(II), Figure S10, Supporting Information) subsequently reacted with H_2_O_2_ via a Russell reaction pathway. Compared with the CNQDs/H_2_O_2_ system (Figure S11, Supporting Information), the greatly obvious triplet peak signal of ^1^O_2_ (α_N_ = 16.9 G, g = 2.0054) in the O‐Fe‐N_4_/H_2_O_2_ system suggested that the Fe species were indispensable for the Russell‐type enzymatic activity (Figure 4d). The specific fluorescent probe singlet oxygen sensor green (SOSG) was used to further determine the generation of ^1^O_2_. As shown in Figure 4e, the mixed solution containing O‐Fe‐N_4_ was detected to have a significantly increased fluorescent intensity with time, compared with the faint fluorescent intensity of the mixed solution without O‐Fe‐N_4_. Notably, the ^1^O_2_‐produce performance was nearly unaffected under hypoxic conditions, suggesting that O‐Fe‐N_4_ could perfectly solve the hypoxia limitation and stimulation dependence of PDT and sonodynamic therapy, microwavedynamic therapy, and radiodynamic therapy. To clarify the mechanism of ^1^O_2_ production, we preadded silver nitrate (e^−^ scavenger), sodium oxalate (h^+^ scavenger), and benzoquinone (BQ, •OOH scavenger) for further characterization. Results proved that the ^1^O_2_ production was significantly inhibited in the presence of sodium oxalate and silver nitrate (Figure S12, Supporting Information), demonstrating that ^1^O_2_ should be generated through the electron transfer from Fe species to H_2_O_2_.^[^ ^27^ ^]^ Intriguingly, the O‐Fe‐N_4_/H_2_O_2_ system also exhibited a considerable characteristic quadruple peak for •OH (α_N_ = α_H_ = 14.9 G, g = 2.0055) at low pH, compared with CNQDs/H_2_O_2_ system at low pH or O‐Fe‐N_4_/H_2_O_2_ system at neutral pH (Figure S13, Supporting Information). Similarly, •OH‐specific fluorescence probe hydroxyphenyl fluorescein (HPF) was employed to identify that •OH was produced due to acid‐mediated Fenton reaction (Figure S14, Supporting Information). Next, the total ROS (^1^O_2_ and •OH) production efficiency was quantitatively in an acid environment via the ROS‐driven 1,3‐diphenylisobenzonfuran (DPBF) degradation. The significant attenuation of DPBF signal intensity was observed in the system containing O‐Fe‐N_4_/H_2_O_2_ (Figure S15, Supporting Information), compared to the acid buffer containing H_2_O_2_ only, while that of CNQDs system displayed no obvious fluctuations (intensity < 3%, Figure S16, Supporting Information) in 30 min. More credible evidence was obtained based on the color reaction between classical probe TMB and ROS (Figure S17, Supporting Information). In the presence of GSH, the generation of ROS was quantified against O‐Fe‐N_4_ concentration, and SA was fitted to be 79.58 U mg^−1^, which was 6‐fold higher than that of O‐Fe‐N_4_ in the absence of GSH (Figure 4f), exhibited competitive catalytic activities than reported Fe‐based SAEs (Table S3, Supporting Information). The above procedure confirmed the Fe(III) species in O‐Fe‐N_4_ played a vital role in self‐cascade enzymatic activity: i.e, depleting GSH and generating ROS (•OH and ^1^O_2_).

### 2.4 Theoretical Calculations of the Reaction Process

**Figure 5:**
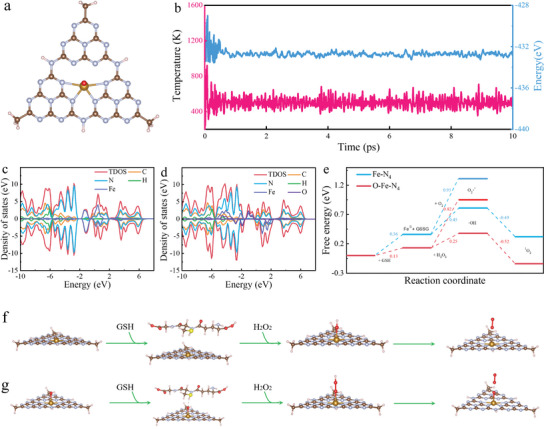
Theoretical studies of O‐Fe‐N_4_ SAEs induced Russell mechanisms. a) The optimized structure model of O‐Fe‐N_4_ moiety. b) Variations of temperature and energy against the time for AIMD simulations of O‐Fe‐N_4_. The simulation is run under 500 K for 10 ps. c,d) Density of states DOS of FeN_4_ moiety (c) and O‐FeN_4_ moiety (d). e) Corresponding free energy diagram. f,g) The proposed catalytic mechanism for Russell reaction on FeN_4_ moiety (f) and O‐FeN_4_ moiety (g).

The O‐Fe‐N_4_ enzyme had more catalytic efficiency and selectivity for ^1^O_2_ production, compared to the Fe‐N_4_ catalyst. To verify that, DFT calculation was performed to reveal the Russell‐type enzymatic mechanism (**Figures** **5**a; Figure S18, Supporting Information):

(1) $$2\;O-\mathrm{Fe}\left(III\right)-N_{4}+2\;\mathrm{GSH}\to 2O-\mathrm{Fe}\left(II\right)-N_{4}+\mathrm{GSSG}$$

(2)

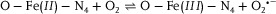

(3) $$O-\mathrm{Fe}\left(II\right)-N_{4}+H_{2}O_{2}\to O-\mathrm{Fe}\left(III\right)-N_{4}{+}^{•}\mathrm{OH}+OH^{-}$$

(4)

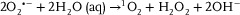




Ab initio molecular dynamics (AIMD) simulations were employed to assess the thermodynamic stability of the O‐Fe‐N_4_ moiety. As illustrated in Figure 5b, the energy remained stable in a numerical range at 500 K and no significant distortion of the geometric structure was checked after 10 ps of simulation, suggesting the thermodynamic stability of O‐Fe‐N_4_ SAEs. The interaction between Fe 3d orbitals and N 2p orbitals is an important factor determining the catalytic performance. The introduction of an axial O atom could break the symmetry of the Fe‐N_4_ configuration (Figures S19 and S20, Supporting Information), and adjust the relative position and electron affinity between Fe and N atoms, which caused the d‐band center of the Fe‐N_4_ moiety to shift toward the Fermi level, thus enhancing the electron attraction, and reducing the energy barriers (Figure 5c,d). Besides, the O‐Fe‐N_4_ moiety tended to promote the reduction of H_2_O_2_ in a slightly acidic environment, which was conducive selectively to conducting the O‐O coupling reaction, producing ^1^O_2_. The O atom engineering increases the catalytic site density of Fe‐N_4_ and makes the reaction activity between the catalyst and substrate more matched, thereby further improving catalytic efficiency. To demonstrate this, the corresponding free energy on O‐Fe‐N_4_ was calculated and compared with that on Fe‐N_4_ (Figure 5e–g). The results illustrated that the activation barrier for the process of Fe(III) underwent reduction to Fe(II) on O‐Fe‐N_4_ was greatly decreased (0.13 eV), suggesting the presence of axial oxygen atom likely stabilized intermediate states by offering additional coordination sites. Charge density difference (CDD) of both moieties with the reaction of GSH were further calculated (Figures S21 and S22, Supporting Information). The introduction of an axial oxygen atom led to increased charge transfer and enhanced interaction between Fe‐N_4_ and GSH, thereby promoting the adsorption of GSH on the surface of Fe‐N_4_. Also, the introduction of an axial oxygen atom improved the adsorption and activation of GSH by increasing the interaction sites with GSH molecules. Specifically, the axial oxygen atom may form hydrogen bonding interactions with groups in GSH, increasing the adsorption sites of GSH molecules and the Fe‐N_4_ surface, thereby enhancing the interaction between them. The above analysis show that the axial oxygen atom played an important role in better adsorption and activation of GSH. Subsequently, H_2_O_2_ underwent the Russell reaction in the presence of the catalyst, resulting in the formation of •OH. Finally, the generated •OH might undergo a series of molecular conformational changes, leading to their conversion into ^1^O_2_. It is worth noting that the energy barrier for the intermediate to react with O_2_ to form O_2_^·−^ was much greater than the free energy to form •OH with H_2_O_2_ (Figure 5e), which proved that the reaction was more likely to selectively produce ^1^O_2_.

### 2.5 O‐Fe‐N_4_ Induces Cell Death

**Figure 6:**
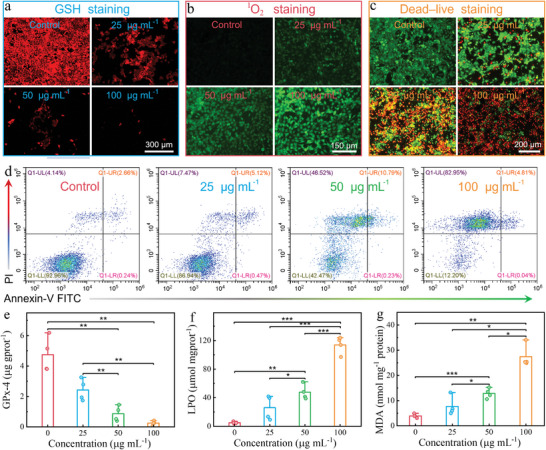
O‐Fe‐N_4_ inhibits cancer cell proliferation. a–c) Intracellular GSH staining (a), ^1^O_2_ staining (b), and Dead‐live staining (c) of the O‐Fe‐N_4_‐treated 4T1 cells with various concentrations (0, 25, 50 and 100 µg mL^−1^). d) Cell death analysis of the O‐Fe‐N_4_‐treated 4T1 cells with various concentrations (0, 25, 50, and 100 µg mL^−1^) by flow cytometry. e–g) GPx‐4 activity (e), LPO level (f), and MDA level (g) measured after treatment with O‐Fe‐N_4_ (*n* = 4). Values are expressed as mean ± standard deviation, ***P < 0.001.

The in vitro GSH depletion potential of O‐Fe‐N_4_ was explored by the GSH assay. The intracellular GSH content and GSH/GSSG ratio tended to attenuate with the increase of O‐Fe‐N_4_ uptake by cells (Figure S23, Supporting Information). Consistent with the GSH assay results, the GSH‐related staining of O‐Fe‐N_4_‐treated 4T1 cells showed a concentration‐dependent performance (**Figure** **6**a). The produced Fe(II) further catalyzed the production of ROS in situ in TME, which was verified by SOSG (Figure 6b) and HPF probe (Figure S24, Supporting Information). The proliferation of malignant tumor cells was significantly inhibited due to the depletion of GSH and the production of highly toxic ^1^O_2_ and •OH. The Cell Titer‐Glo Luminescent assay showed the survival rate of malignant tumor cells was only 19% when they were incubated with O‐Fe‐N_4_ at 100 µg mL^−1^ in a slightly acid medium for 24 h (Figure S25, Supporting Information). Calcein‐AM and PI co‐staining were employed to examine the antiproliferation effect of the O‐Fe‐N_4_ SAEs and the predominant red fluorescence signal was detected when the concentration of nanoadjuvant was 100 µg mL^−1^ (Figure 6c), suggesting the enzymatic behavior of O‐Fe‐N_4_ in response to TME. On the contrary, the 4T1 cells treated with CNQDs in an acidified medium exhibited negligible damage effect (Figure S26, Supporting Information), implicating the Fe signal from O‐Fe‐N_4_ was a key factor in inhibiting the proliferation of cancer cells. Additionally, the flow cytometry analysis with the FITC‐PI staining kit further proved that O‐Fe‐N_4_ SAEs could increase the necrosis rate of tumor cells (Figure 6d; Figure S27, Supporting Information).

The underlying mechanism of O‐Fe‐N_4_‐induced tumor cell death was further investigated. As envisaged, the GPx‐4 level within tumor cells significantly decreased as the upswing of O‐Fe‐N_4_ concentrations (Figure 6e), further indicating the remarkable GSHOD‐like performance of O‐Fe‐N_4_. The consumption of GSH could downregulate reductive substances in TME, amplifying oxidative stress and inducing LPO. The LPO‐sensitive probe BODIPY^493/503^ was used to check the LPO level in 4T1 cells. Predictably, our results showed that the LPO content increased with the enhanced O‐Fe‐N_4_ dosages (Figure 6f), suggesting the O‐Fe‐N_4_ SAEs mediated 4T1 cell death via ferroptosis. In addition, the levels of malondialdehyde (MDA, Figure 6g) and 4‐hydroxynonenal (4‐ HNE, Figure S28, Supporting Information) in 4T1 cells, were linearly correlated with the incubation concentration of O‐Fe‐N_4_, further implying the ferroptosis. Furthermore, the intracellular LPO level was checked by the LPO‐sensitive dye C11‐BODIPY. The strong green fluorescence could be observed in O‐Fe‐N_4_‐treated cancerous cells, while the positive signal decreased in 4T1 cells with Ferrostatin‐1 (Fer‐1, a ferroptosis inhibitor) pre‐treatment (Figure S29, Supporting Information), confirming that the O‐Fe‐N_4_ SAEs could cause ferroptosis. Furthermore, mitochondrial membrane potential impairment was demonstrated by JC‐1 staining (Figure S30, Supporting Information), indicating that ferroptosis leads to mitochondrial dysfunction, which could be verified by Bio‐TEM (Figure S31, Supporting Information). In addition, mitochondrial membrane potential damage (Figure S30, Supporting Information) and intracellular ^1^O_2_ production capacity were inhibited (Figure S32, Supporting Information) when cancer cells were pre‐treated with Fer‐1 reagent, reconfirming that the anti‐cancer effect of O‐Fe‐N_4_ depends on ferroptosis pathway. Interestingly, western blotting (WB) showed that the expression levels of PARP, Caspase‐3, Bax, Bcl‐2, and Bcl‐xl fluctuate as the upswing of O‐Fe‐N_4_ dosage (Figure S33, Supporting Information), suggesting the apoptosis was involved in the process of tumor cell death. Moreover, the results of the WB assay showed the obvious downregulation of GPx‐4 level in 4T1 cells, which was attributed to the GSH consumption‐ability of O‐Fe‐N_4_. The ROS storm and the reduced activity of GPx‐4 could cause irreversible LPO in cancer cells, which was reconfirmed by the WB assay (Figure S33, Supporting Information). Collectively, these findings confirmed that O‐Fe‐N_4_ with cascading enzymatic properties could lead to tumor cell damage through apoptosis, necrosis, and ferroptosis pathways.

### 2.6 Security Verification of O‐Fe‐N_4_

The O‐Fe‐N_4_ enzyme failed to induce a significant decrease in cell viability in a normal medium (Figure S34, Supporting Information). In vivo distribution investigation (Figure S35, Supporting Information) and the blood half‐life (1.77 h, Figure S36, Supporting Information) implied the Fe signal intensity gradually weakened over time, demonstrating efficient excretion of O‐Fe‐N_4_. Moreover, the signal intensity in the tumor became elevated (4.84%) at 24 h post‐injection. No significant fluctuation in the hematological markers of the mice receiving the O‐Fe‐N_4_ procedure pointed out that the liver function and various physiological indicators were normal (Figures S37–S39, Supporting Information). In addition, a 15‐day histological analysis revealed no obvious abnormalities or morphological lesions (Figure S40, Supporting Information). All the above data indicated that the O‐Fe‐N_4_ adjuvant had negligible off‐target toxicity.

### 2.7 The Tumor Inhibition Ability of O‐Fe‐N_4_ In Vivo

**Figure 7:**
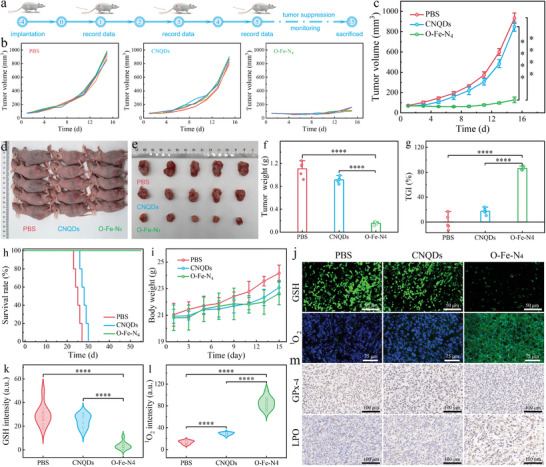
Catalytic tumor regression by O‐Fe‐N_4_. a) Diagram of in vivo treatment. b,c) Tumor proliferation curves. d,e) Digital photos of mice (d) and tumor (e) from PBS, CNQDs, and O‐Fe‐N_4_ groups at day 15. f) Tumor weight of dissected tumors. g) TGI rates treated with CNQDs or O‐Fe‐N_4_ group at day 15 in contrast to PBS group. h) Survival percentages of the mice. i) Body weight curves in different treatment groups. j) Immunofluorescence staining. k,l) GSH intensity (k), ^1^O_2_ intensity (l) quantified from 10 random fields of view selected from each group. m) Immunohistochemical characterization. Values are expressed as mean ± standard deviation, ****P < 0.0001.

The GSH consumption and ROS accumulation within malignant tumors mediated by self‐cascade catalytic performances of the O‐Fe‐N_4_ cargo with negligible off‐target toxicology encouraged us to create further in vivo model (**Figure** **7**a). When the average size of tumors reached 70 mm^3^, mice were randomly assigned to three groups and treated with: 1) PBS (control); 2) CNQDs; and 3) O‐Fe‐N_4_. Almost no suppression ability in tumor growth was found in mice administrated with PBS and CNQDs during the observation period (Figure 7b,c). Notably, the tumor proliferation of mice in the O‐Fe‐N_4_‐treated group was obviously inhibited (Figures 7b–f; Figure S41, Supporting Information), and the tumor growth inhibition (TGI) rate was measured to be 86.1% (Figure 7g), suggesting again the Fe species played a key role in tumor‐specific self‐cascade enzymatic therapy. Due to the considerable tumor inhibitory ability, the survival period of O‐Fe‐N_4_‐treated mice reached over 55 days (Figure 7h). In addition, the average body weight showed an upward trend during the treatment period, validating the satisfactory biocompatibility of O‐Fe‐N_4_ (Figure 7i).

The relevant mechanisms involved in tumor suppression were further assessed via immunofluorescence, immunohistochemical, and WB assay. Compared with the control group and the CNQD‐treated group, the dissected tumors of the O‐Fe‐N_4_‐treatment group showed significantly mitigated GSH expression, enhanced ^1^O_2_ and •OH expression, and obvious nuclear structure damage (Figure 7j; Figure S42, Supporting Information). The positive rate expression of relevant assays further verified that the O‐Fe‐N_4_ SAEs overwhelmed the redox homeostasis (Figure 7k), as well as boosted ROS accumulation (Figure 7l) in the slightly acidic tumor tissue, thereby inhibiting the proliferation of malignant tumors. Immunohistochemical characterization further revealed that downregulation of GPx‐4 activity and LPO accumulation occurred in 4T1 tumor tissue (Figure 7m), which verified the occurrence of ferroptosis in the tumor. Moreover, the WB assay of isolated tumors revealed the O‐Fe‐N_4_‐treatment group had LPO‐induced ferroptosis and apoptosis, in contrast to the PBS‐ and CNQD‐treated groups (Figure S43, Supporting Information). Collectively, these findings confirmed that the O‐Fe‐N_4_ SAEs could serve as a promising agent to repress tumor proliferation through ferroptosis and apoptosis.

## 3 Conclusions

In summary, we have demonstrated an ultrasmall carbon nitride quantum dot‐supported iron‐based SAEs with axial O atom engineering Fe‐N_4_ moieties (O‐Fe‐N_4_). The O‐Fe‐N_4_ enzyme possesses a surprising sequential catalytic ability to induce ^1^O_2_ and •OH production with an SA value of 79.58 U mg^−1^ at pH 6.2, which surpasses that of so‐far‐reported Fe‐N_4_ catalysts. The superior enzymatic performance originates from higher affinity, better activation barrier, and stronger selectivity of Fe‐N_4_ moieties brought by axial oxygen atoms. In vivo and in *vitro* investigations show that GSHOD‐like activity of O‐Fe‐N_4_ can lead to GPx‐4 activity downregulation, and POD‐mimic ability can produce highly efficient ROS, and both synergistically make tumor cells undergo ferroptosis, apoptosis, and necrosis. The O‐Fe‐N_4_ SAEs not only addresses the inherent limitations of the ^1^O_2_‐elevated tumor therapy strategy but also provides valuable insights into the advanced catalytic efficiency of Fe‐N_4_ catalysts.

## 4 Experimental Section

### Catalyst Preparation

30 mL formamide solution containing FeCl_2_ (0.380 g, 3 mmol) was stirred at 500 rpm till completely dissolved. Subsequently, the mixture was transferred into a 50 mL Teflon‐lined stainless‐steel autoclave (Anhui Kemi Instrument Co., Ltd). The sealed vessel was heated at 200 °C for 24 h before it was cooled to room temperature. The supernatant was collected by centrifugation (10 000 rpm, 10 min) and dialyzed for 24 h to remove unreacted precursors. Subsequently, the aqueous dispersion was purified via silica column chromatography using ethanol as the eluent. Finally, centrifuged at 12 000 rpm and the precipitate was washed with deionized water 3 times, and then dried in a vacuum oven at 50 °C.

## Conflict of Interest

The authors declare no conflict of interest.

## Supporting information

Supporting Information

## Data Availability

The data that support the findings of this study are available from the corresponding author upon reasonable request.
