# Supplementary material for: Axial O Atom‐Modulated Fe(III)‐N4 Sites for Enhanced Cascade Catalytic 1O2‐Induced Tumor Therapy
Source: Adv Sci (Weinh). 2024 Jul 1;11(33):2307254. doi: 10.1002/advs.202307254 (PMC11434021; doi:10.1002/advs.202307254)
Supplement: Supplementary file 1 — Supporting Information [file ADVS-11-2307254-s001.docx]

Supporting Information

Axial O Atom-Modulated Fe(Ⅲ)-N_4_ Sites for Enhanced Cascade Catalytic ^1^O_2_-Induced Tumor Therapy

Hongji Liu,^#^ Biao Yu,^#^ Pengqi Yang, Yang Yang, Zhiming Deng, Xin Zhang,* Kai Wang,* Hui Wang*

1. **General Experimental Methods**

**1.1 Materials:** Iron(Ⅱ) chloride (FeCl_2_), formamide (Ar, 99%), ethanol (99.7%), 5,5-dimethyl-1-pyrroline N-oxide (DMPO), 2,2,6,6-Tetramethylpiperidine (TEMP), 3,3’,5,5’-tetramethylbenldlne (TMB), 1,3-diphenylisobenzonfuran (DPBF), dimethyl sulfoxide (DMSO), sodium acetate (NaAc), acetic acid (HAC), hydrogen peroxide (30% (wt/vol), H_2_O_2_), glutathione (GSH), sodium hydroxide, 4’,6-diamidino-2-phenylindole (DAPI) were purchased from Aladdin and were used as received without further purification. The water used in all experiments was of Millipore Milli-Q grade.

**1.2 Characterization:** The XRD, Raman, UV-vis spectra, XPS, PL spectra, and Fourier transform infrared were performed using D8 Bruker X-ray diffractometer, Horiba Jobin YvonT64000 Micro-Raman instrument, Rayleigh UV-2601 ultraviolet-visible-NIR spectrophotometer, ESCALAB 250 X-ray photoelectron spectrometer, Horiba Jobin Yvon Co. Fluorolog-3-21 spectrofluorometer, and Bruker Vertex 80V FT-IR spectrometer, respectively. The EPR spectra and vacancy structure were recorded on Bruker EMX plus 10/12 spectrometer. All the NMR experiments were performed at the High Magnetic Field Laboratory, Chinese Academy of Sciences. The morphology of O-Fe-N_4_ was measured on a Talos F200X TEM (FEI) operated at 200 keV. Aberration-corrected STEM imaging and electron energy-loss spectroscopy (EELS) were performed on the Nikon HERMES-100 instrument equipped with a C3/C5 corrector, operated at 60 kV with a convergence angle of around 32 mrad and a collection angle of about 75 mrad.

**1.3 XAFS Measurements:** The X-ray absorption spectra including XANES and EXAFS spectra of O-Fe-N_4_ were collected at the Singapore Synchrotron Light Source (SSLS) center (operating at 2.5 GeV with an average electron current of below 200 mA). The Fe K-edge XANES data was recorded in the transmission mode, and Fe foil, Fe_2_O_3_, and FePc were used as references. EXAFS data was extracted and processed based on the standard procedures using the ATHENA module implemented in the IFEFFIT software packages. The k^3^-weighted Fourier transform (FT) of χ(k) in R space was obtained in the range of 0-14.0 Å^-1^ by applying a Bessel window function.

**1.4 GSHOD-Mimicking Activity of O-Fe-N_4_:** The O-Fe-N_4_ solution (5 mg mL^-1^, 10 mL) was mixed with GSH solution (5 mM, 10 mL) for various times. The supernatants were collected by centrifugation for ^1^H NMR testing or GSH kit (Beijing Solarbio Science & Technology Co., Ltd) detection, respectively.

**1.5 The SA Value of GSHOD-like Activity:** GSH solution was mixed with O-Fe-N_4_ with various concentrations, followed by the detection of the absorbance of DTNB at 412 nm for fitting with a linear function.

**1.6 GSH Reduction-Mediated ^1^O_2_ or •OH Generation of O-Fe-N_4_:** A mixed solution of O-Fe-N_4_ solution (5 mg mL^-1^) with GSH (5mM) was prepared. SOSG (10 μl; from SOSG assay kit for ^1^O_2_) or HPF (10 μl; from HPF assay kit for •OH), 200 μl of H_2_O_2_ (10 mM), and 400 μl of the mixture was added with 1.4 ml of PBS buffer. The change of SOSG or HPF fluorescence intensity was monitored by the fluorescence spectrometer with excitation/emission of 504/525 nm or 490/515 nm, respectively.

**1.7 Characterization of ROS Mediated by O-Fe-N_4_ *via* DPBF:** 2 mL of O-Fe-N_4_ solution with variable concentration (0 ~ 2.4 µg mL^-1^) were mixed with 50 μL DPBF (10 mM, solution in DMSO) in pH 6.2. The absorption intensity of the mixed solutions at 421 nm was detected by a UV spectrophotometer after adding H_2_O_2_ (100 µL) to the above solution.

**1.8 The SA Value of Enzyme-Mimic of O-Fe-N_4_ with or without GSH:** A mixed solution of 2 mL of O-Fe-N_4_ with various concentrations, TMB (10 mg mL^-1^, 200 μL), and H_2_O_2_ (200 μL) was prepared, followed by the detection of the absorbance of oxidized TMB at 650 nm for fitting with a linear function. The SA value of the enzyme-like activity of O-Fe-N_4_ containing GSH is measured only by adding 5 mM GSH to the mixture, and other test methods are the same.

**1.9 DFT Calculation:** We used the DFT as implemented in the Vienna Ab initio simulation package (VASP) in all calculations. The exchange-correlation potential is described by using the generalized gradient approximation of Perdew-Burke-Ernzerhof (GGA-PBE). The projector augmented-wave (PAW) method is employed to treat interactions between ion cores and valence electrons. The plane-wave cutoff energy was fixed to 500 eV. Given structural models were relaxed until the Hellmann–Feynman forces smaller than -0.02 eV/Å and the change in energy smaller than 10^-5^ eV was attained. During the relaxation, the Brillouin zone was represented by a Γ centered k-point grid of 6×6×1. Grimme’s DFT-D3 methodology was used to describe the dispersion interactions among all the atoms in adsorption models. Employing the climbing image nudged elastic band method (CI-NEB), we computed the minimum energy pathway of the cyclization reaction along with its corresponding activation barrier.

**1.10 *In Vitro* GSH Staining**: The 4T1 cells were cultured in 6-well plates containing coverslips bottom at 3 × 10^5^ cell mL^-1^ and incubated with different concentrations of O-Fe-N_4_ (0, 25, 50, and 100 μg mL^-1^) for 12 h. The 4T1 cells and incubated with GSH antibody overnight at 4 ℃. The samples were then washed three times and then incubated with a secondary antibody for 2 h. They were then washed in PBS as before followed by incubation with DAPI diluted in PBS for 5 min. Samples were mounted in an anti-fading reagent. The cell imaging was performed by confocal microscope (Olympus, Japan). In addition, GSH and GSSG contents of cells were measured according to the manufacturer’s instructions.

**1.11 Intracellular ^1^O_2_ or •OH Staining:** The 4T1 cells in 12-well plates (3 × 10^5^ cells mL^-1^) were cultured in RPIM1640 containing 10% FBS at pH 6.2, while treated with O-Fe-N_4_ (0, 25, 50, and 100 μg mL^-1^) for 12 h. Subsequently, the cells can incubate with 10 μM SOSG or HPF for 15 min, resulting in fluorescent imaging that can be identified by the EVOS FL imaging system (Life Technologies, USA).

**1.12 Calcein-AM/PI Staining and** **Annexin V-FITC/PI Assay**: The Calcein-AM (emitted green fluorescence) and PI (emitted red fluorescence) staining was used to distinguish between dead and live cells. The 4T1 cells were cultured in 12-well plates at 3 × 10^5^ cells per well and incubated with different concentrations of O-Fe-N_4_ (0, 25, 50, and 100 μg mL^-1^) in pH 6.2 for 24 h. Then, 4T1 cells were stained with Calcein-AM/PI for 15 min. The cell imaging was performed by confocal microscope (Olympus, Japan). For Annexin V-FITC/PI assay, cells were detected by flow cytometry using the annexin V/PI assay kit following the manufacturer's instructions and data analysis was analyzed using Flow Jo software.

**1.13 Intracellular LPO Measurement:** A BODIPY^493/503^ probe was used to investigate the production of LPO in O-Fe-N_4_-treated 4T1 cells. Briefly, 3 × 10^5^ cells per well were seeded in the 12-well plates. After reaching 80% confluence, cells were incubated with O-Fe-N_4_ (0, 25, 50, and 100 μg mL^-1^) for 12 h. After removing the medium, cells were washed with PBS three times, and stained with BODIPY^493/503^ (2 µm, in free medium) for 30 min. After washing with PBS three times, cells were collected and subjected to flow cytometry or identified by confocal microscopy (Olympus, Japan).

**1.14 Enzymes Activity Assay:** The cell levels of MDA and 4-HNE were assayed using commercial standard kits (Aimeng Youning, Shanghai, China). The absorbance rates were recorded by a spectrophotometer at 450 nm for both MDA and 4-HNE. The results of MDA and 4-HNE are reported in nmol/mg protein, and ng/mg protein respectively. Furthermore, 4T1 cells in a 12-well plate were treated in a similar procedure. The cell levels of GPx-4 and LPO were assayed according to the manufacturer’s instruction on Enzyme activity analysis (Nanjingjiancheng, Nanjing, China). The results of GPx-4 and LPO are reported in µg/g protein, and µmol/g protein respectively. The protein content was assayed by a BCA protein quantification kit (Vazyme Biotech Co., Ltd, Nanjing, China).

**1.15 Analysis of the Change of Mitochondrial Membrane Potential:** After cell attachment, O-Fe-N_4_ (50 μg mL^-1^) were added for 24 hours in pH 6.2. After being stained with JC-1 and Hoechst 33342 (10 μM) according to the manufacturer’s protocols, the cells were imaged with a fluorescence confocal microscope. To examine the effects of ferroptosis inhibitor, Fer-1 (10 μM) was added to the 96-well plates 2 hours before co-incubation with O-Fe-N_4_ at concentration of 50 μg mL^-1^, followed by the aforementioned procedures.

**1.16 Western Blots Analysis:** The 4T1 cells were seeded into 12-well plates and allowed to adhere overnight. 4T1 cells were incubated with PBS or O-Fe-N_4_ with various concentrations at pH 6.2 for 24 h. The cells were lysed by M-Per^TM^ Mammalian Protein Extraction Reagent (Thermo Scientific, Waltham, MA, USA) at 4 °C for 20 min. The cell lysate was collected and added to a 5 × SDS loading buffer. The samples were denatured at 95 °C for 8 min. The proteins were separated by SDS-PAGE gels, transferred onto nitrocellulose membranes, incubated with 5% skimmed milk, and then incubated with primary antibodies at 4 °C overnight and then incubated for 1 h at room temperature with corresponding secondary antibodies. Image results were obtained by Bio-Rad ChemiDoc TM XRS+ System and Beijing Tanon Fine-do X6.

**1.17 Cell Viability Assay**: 4T1 cells were plated in 96-well plates with 3000 cells per well and culture under a 5% CO_2_ atmosphere in RPMI-1640 medium for 36 h before incubation with various concentrations of O-Fe-N_4_ or PBS. Cell viability assays were performed using the Cell Titer-Glo Luminescent assay (Promega, Madison, WI, USA). The Luminescence was measured in a multilabel plate reader (Envision PerkinElmer, USA). The fluorescence intensity of PBS-treated cells was defined as 100%.

**1.18 Animal Model**: All animal welfare and experimental procedures were performed strictly according to the guidelines from the National Institutes of Health Guide for the Care and Use of Laboratory Animals (NIH Publication, 8th Edition, 2011, USA). Additionally, all *in vivo* experiments complied with the Guidelines of the Institutional Animal Care and Use Committee of Hefei Institutes of Physical Science, Chinese Academy of Sciences that approved all protocols (Animal Protocol Form # DWLL (P)-2023-91), and all efforts were made to minimize suffering. Female nude mice at six weeks old (GemPharmatech Co. Ltd, China) to use in tumor model through subcutaneous injection of 4T1 cells suspended in Matrigel into the right flanks. The bearing 4T1 tumor nude mice were treated with different procedures when the tumor grew to the fourth day (~ 70 mm^3^). Animal experiments were carried out according to a protocol approved by Institutional Animal Care and Use.

**1.19 Hematology and Tissue Histopathological Examinations****:** Nude mice were injected with O-Fe-N_4_ (10 mg kg^-1^) or PBS (200 μL, control) through the tail vein for various times (7^th^ d and 15^th^ d), and then blood and serum were collected for analysis. The blood biochemical analysis was tested using a microplate reader (Rayto, China) and a fully automatic biochemical analyzer (Rayto, China). Major organs were collected on the 15^th^ day and were embedded in paraffin wax, after being fixed in 4% paraformaldehyde for 48 h. Slides were scanned using an Aperio Scanner.

**1.20 *In Vivo* Therapeutic Evaluation of O-Fe-N_4_:** The tumor-bearing mice were injected with O-Fe-N_4_ (10 mg kg^-1^), or CNQDs (10 mg kg^-1^) or the same volume of PBS through the tail vein, respectively. The same dose of O-Fe-N_4_ or CNQDs or PBS was injected at the same time point 2 days and 4 days later. Monitor the length (a) and width (b) of the tumor during the treatment, and calculate the tumor size by formula: Volume = ab^2^/2.

**1.21 Tumor GSH Staining:** Tumor tissues were subjected to permeabilization and blocking (30 min) in TBS + 0.3% BSA (bovine serum albumin) + 0.3% Triton X-100 at RT. Subsequent operations were performed as described in the section on *in vitro* GSH staining.

**1.22 Tumor ROS Staining**: The tumors were sliced after freezing (−20 °C), fixing (acetone), and washing (PBS). The sections were then incubated with SOSG or HPF (10 min) before imaging.

**1.23 Tumor TUNEL Staining**: The operation process of the TUNEL analysis was the same as that of the H&E analysis, except that the staining was replaced with a TdT-mediated dUTP Nick-End Labeling kit (TUNEL).

**1.24 Tumor H&E Staining**: The H&E analysis was performed as described in the aforementioned section.

**1.25 Tumor WB Assay:** The WB analysis was performed as described in the aforementioned section.

**1.26 Statistical Analysis:** Data were expressed as mean ± SD. The statistical significance of the data was compared by Student’s two-tailed t-test. Analysis of variance (ANOVA) was used to analyze the differences among the different groups using software Microsoft Excel. The differences were considered to be significant when p < 0.05.

1. **Supplementary Figures**

**Figure S1.** The Raman spectrum of O-Fe-N_4_.

**Figure S2.** XPS survey of O-Fe-N_4_.

**Figure S3.** XPS high-resolution survey scan of Fe 2p in O-Fe-N_4_.

**Figure S4.** XANES linear combination fitting analysis of O-Fe-N_4_, the best-fit parameters are shown in Table S1.

**Figure S5.** XPS high-resolution survey scans of C1s in O-Fe-N_4_.

**Figure S6.** The corresponding fitting curve of the EXAFS spectrum of O-Fe-N_4_ at k space.

**Figure S7.** Fitting curves of the EXAFS of Fe foil in the R-space and k-space, respectively. EXAFS fit was carried only for the first coordination shell. The measured and calculated spectra are well-matched, and the best-fit parameters are shown in Supplementary Table 2.

**Figure S8.** Fitting curves of the EXAFS of Fe_2_O_3_ in the R-space and k-space, respectively. The measured and calculated spectra are well-matched, and the best-fit parameters are shown in Supplementary Table 2.

**Figure S9.** Fitting curves of the EXAFS of FePc in the R-space and k-space, respectively. The measured and calculated spectra are well-matched, and the best-fit parameters are shown in Supplementary Table 2.

**Figure S10.** Changes in contents of Fe(Ⅲ) and Fe(Ⅱ) over time (*n* = 3). The valent state of Fe was determined *via* chromogenic agents as follows. After the reaction of O-Fe-N_4_ and GSH for different time periods, the precipitates were obtained by high-speed centrifugation and then digested with aqua regia overnight to ionize the metal active sites before the colorimetric assay. **Fe^Ⅱ^:** the mixture (100 μL) was added into the 900 μL aqueous solution of 1,10-phenanthroline monohydrate (2 mM), then its concentration is quantified by checking the absorbance at λ=510 nm; **Fe^Ⅲ^:** the the mixture of 100 μL was added into the 900 μL phosphate buffer solution of KSCN (pH=6, the concentration of phosphate is 7 mM, and the concentration of KSCN is 10 mM), then the concentration of Fe^Ⅲ^ is quantified by checking the absorbance at λ=411 nm. Values are expressed as mean ± standard deviation.

**Figure S11.** The EPR signals of ^1^O_2_ generation from CNQDs/H_2_O_2_ system in pH 6.2.

**Figure S12.** The ^1^O_2_ generation rate in the presence of various ROS scavengers, respectively. The ESR signal intensity of the O-Fe-N_4_/H_2_O_2_ system without ROS scavenger was defined as 100% (*n* = 3). Values are expressed as mean ± standard deviation.

**Figure S13.** The EPR spectra of •OH in different systems with DMPO as the trapping agent.

**Figure S14.** Time-dependent •OH generation under acidified PBS conditions detected by HPF (*n* = 3). Values are expressed as mean ± standard deviation.

**Figure S15.** The ROS generation efficiency in O-Fe-N_4_/DPBF/H_2_O_2_ systems with various concentrations of O-Fe-N_4_.

**Figure S16.** The ROS generation efficiency in CNQDs/DPBF/H_2_O_2_ systems in pH 6.2.

**Figure S17.** The UV-Vis-NIR spectra of the mixed solution with different systems in the acid medium.


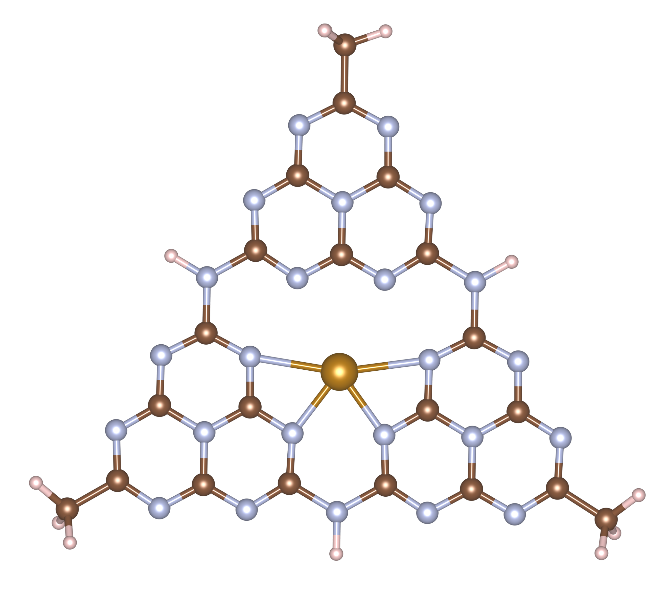


**Figure S18.** The optimized structure model of O-Fe-N_4_ moiety.


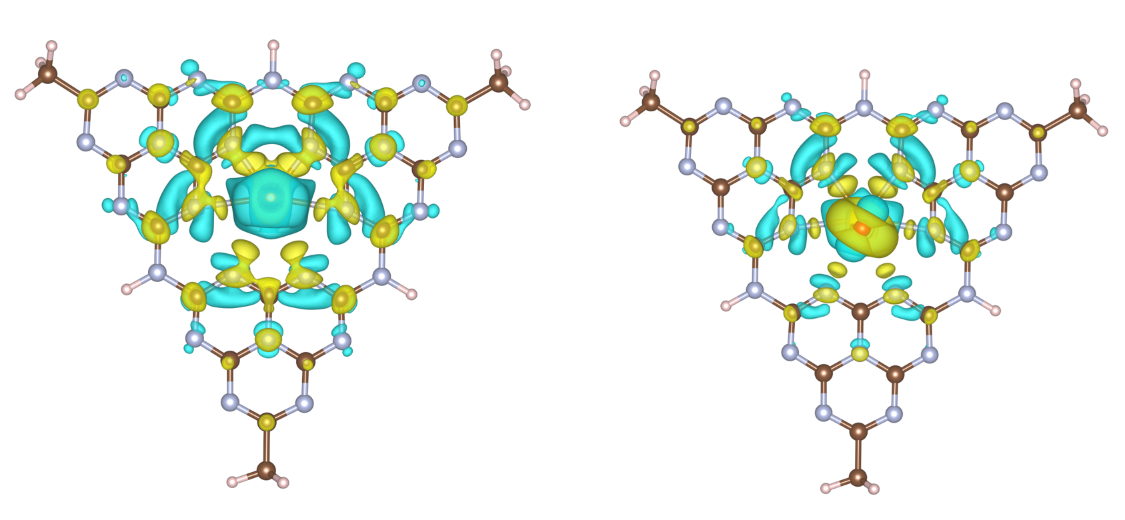


**Figure S19.** Top view of the charge densities of Fe-N_4_ model (left) and the O-Fe-N_4_ model (right), respectively.


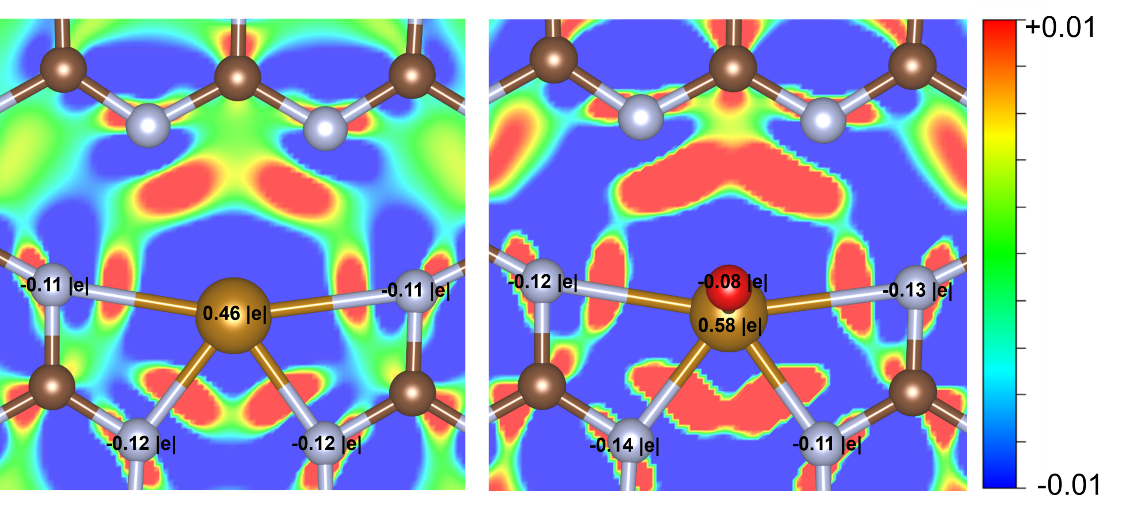


**Figure S20.** Bader charge distribution of Fe-N_4_ model (left) and O-Fe-N_4_ model (right) , respectively.


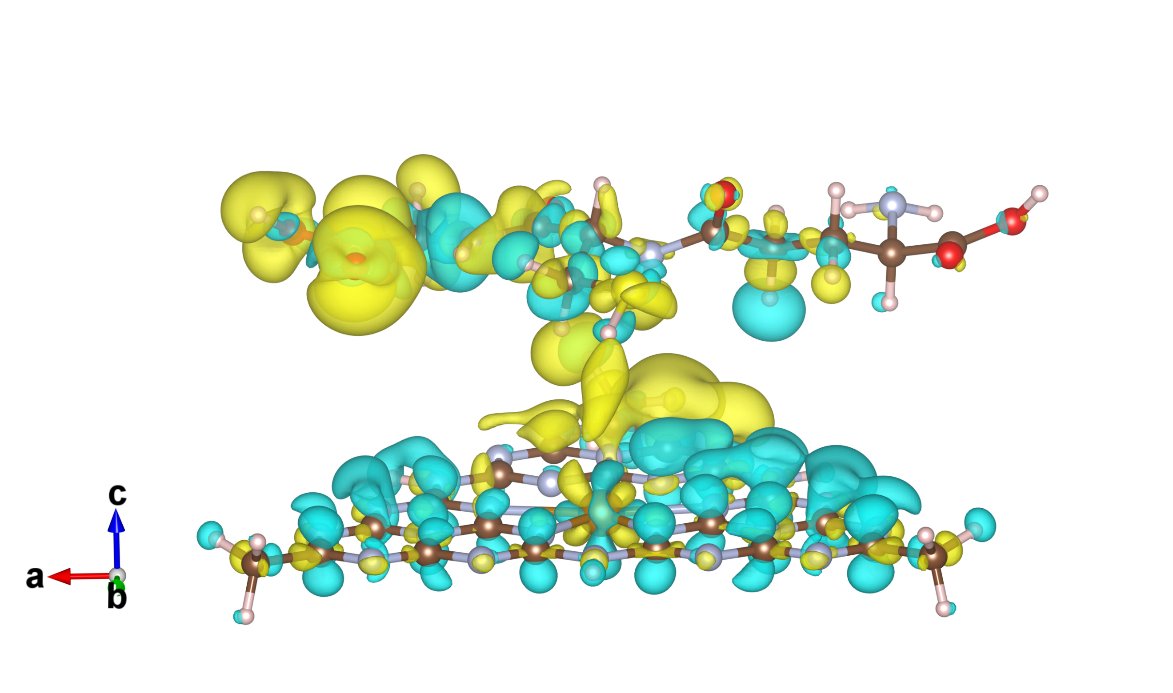


**Figure S21.** Difference charge density of Fe-N_4_ moiety with the adsorption of GSH, where the positive and negative charges are shown in yellow and cyan, respectively.


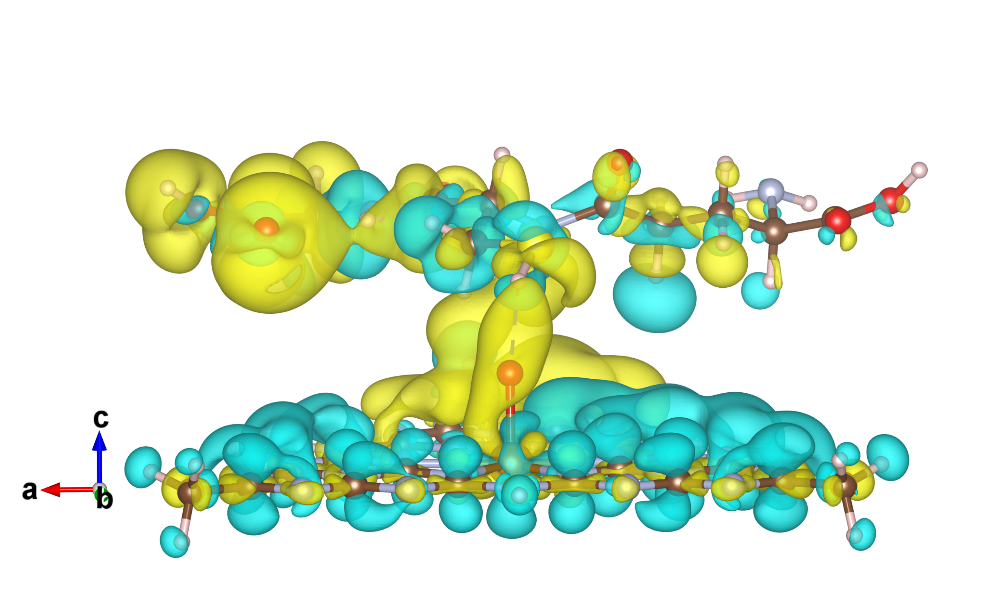


**Figure S22.** Difference charge density of O-Fe-N_4_ moiety with the adsorption of GSH, where the positive and negative charges are shown in yellow and cyan, respectively.

**Figure S23.** (a) Depletion of GSH in tumor cells. (b) GSH/GSSG level in 4T1 cells after the O-Fe-N_4_ SAEs treatment (*n* = 3). Values are expressed as mean ± standard deviation. ***P < 0.001.


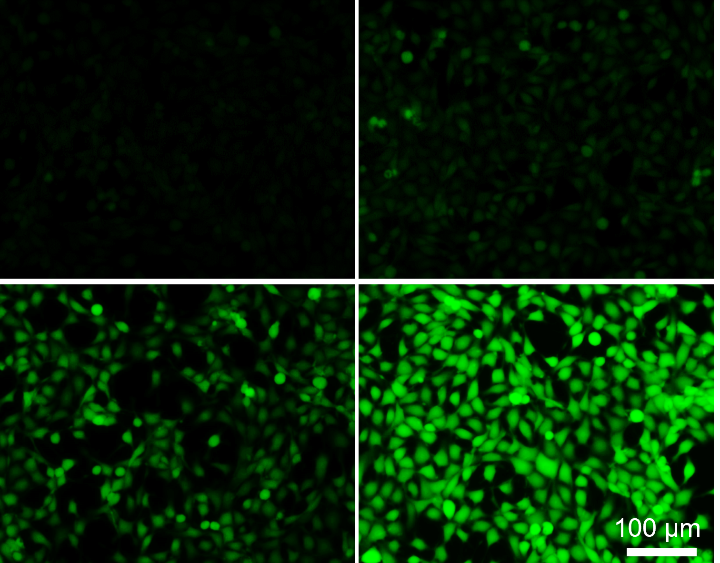


**Figure S24.** •OH staining of the O-Fe-N_4_-treated 4T1 cell with various concentrations (0, 25, 50 and 100 μg mL^-1^).

**Figure S25.** Relative cell viability assays of O-FeN_4_ under acidified culture medium (*n* = 6). Values are expressed as mean ± standard deviation.

**Figure S26.** Relative cell viability assays of CNQDs under acidified culture medium (*n* = 6). Values are expressed as mean ± standard deviation.

**Figure S27.** The corresponding quantitative analysis of apoptosis measured by flow cytometry based on Annexin V-FITC and PI staining of 4T1 cells after the various treatments.

**Figure S28.** 4-HNE levels measured after treatment with O-Fe-N_4_. Values are expressed as mean ± standard deviation. ***P < 0.001.


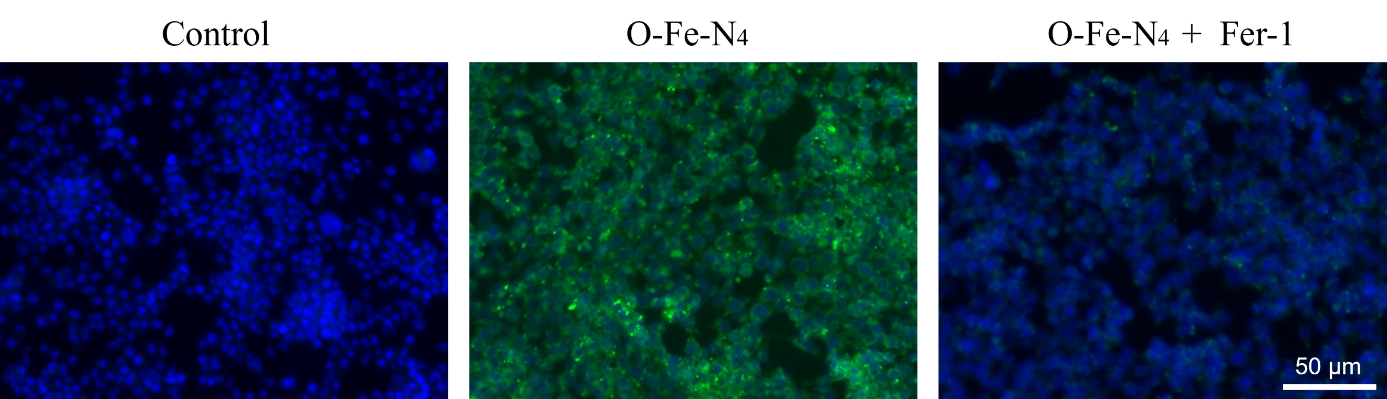


**Figure S29.** C11-BODIPY staining of 4T1 cells with various trentment. To investigate the underlying mechanisms of cell death, 4T1 cells were preincubated with Fer-1 for 2 h under a weak acid condition, and subsequently treated with O-Fe-N_4_ for 24 h.


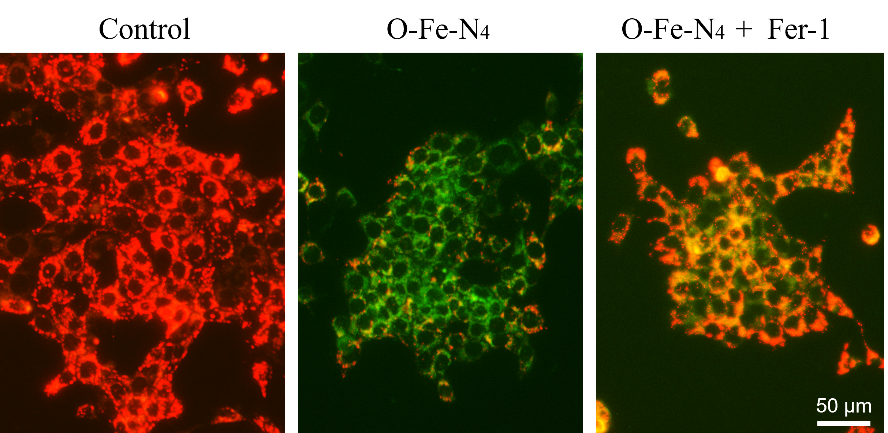


**Figure S30.** JC-1 staining of 4T1cells with various trentment.

**
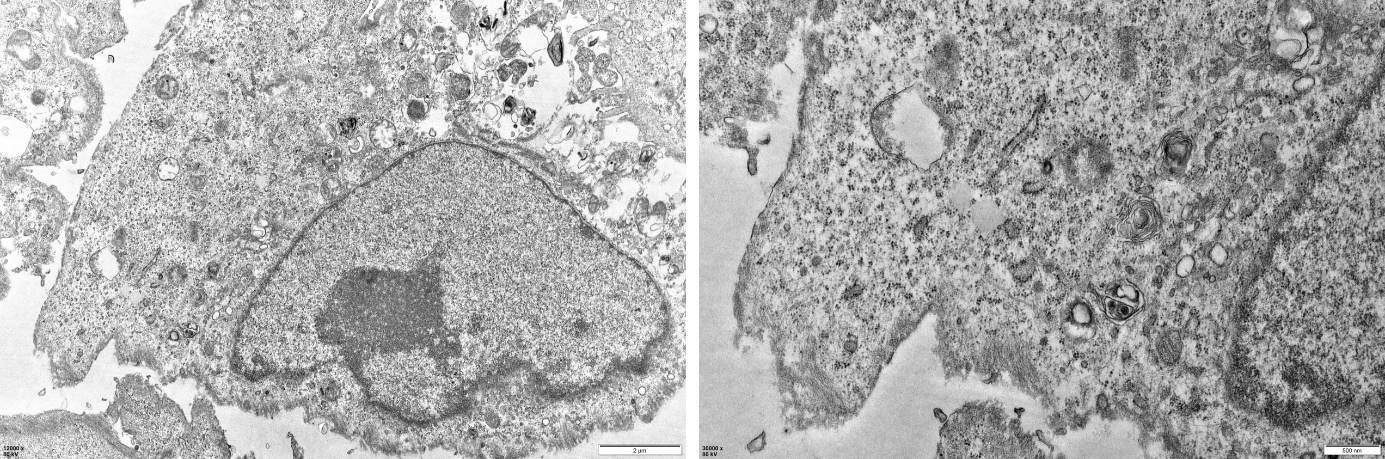
**

**Figure S31.** Representative Bio-TEM images of 4T1 cells show cellular morphology changs after O-Fe-N_4_ treatment. The cells showed an irregular nuclei morphology, broken and blebbed membrane, and noticeable mitochondria shrinkage.


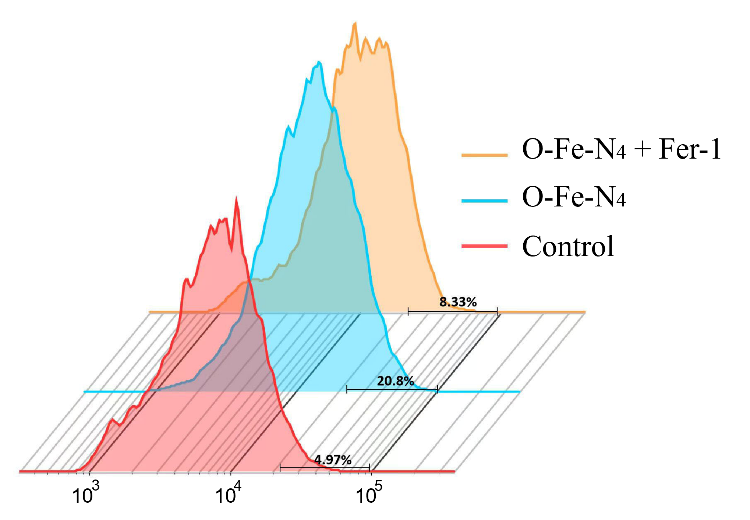


**Figure S32.** Flow cytometry assay showed the O-Fe-N_4_ SAEs induced the production of ^1^O_2_ in 4T1 cells.

**
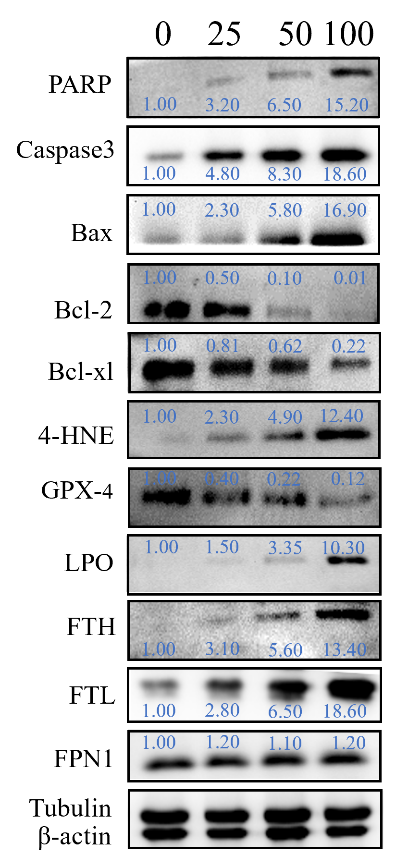
**

**Figure 33.** Western blotting assay of the representative protein expression after treatments with PBS or O-Fe-N_4_ with various doses.

**Figure S34.** Relative cell viability assays of O-Fe-N_4_ under a neutral culture medium (*n* = 6). Values are expressed as mean ± standard deviation.

**Figure S35**. *In vivo* Fe biodistributions after intravenous injections of O-Fe-N_4_ for 6, 12, 24, and 48 h (*n* = 3). Values are expressed as mean ± standard deviation.

**Figure S36.** Blood circulation curve of Fe concentration after intravenous administration of O-Fe-N_4_. The blood-circulation half-time was calculated to be 1.77 h (*n* = 3). Values are expressed as mean ± standard deviation.

**Figure S37.** Hematological index: alanine aminotransferase (ALT), aspartate aminotransferase (AST), and alkaline phosphatase (ALP) (*n* = 3). Values are expressed as mean ± standard deviation.

**Figure S38.** Hematological index: hemoglobin (HGB), mean corpuscular volume (MCV), and hematocrit (HCT) (*n* = 3). Values are expressed as mean ± standard deviation.

**Figure S39.** Hematological index: Urea, red blood cells (RBC), and white blood cells (WBC) (*n* = 3). Values are expressed as mean ± standard deviation.


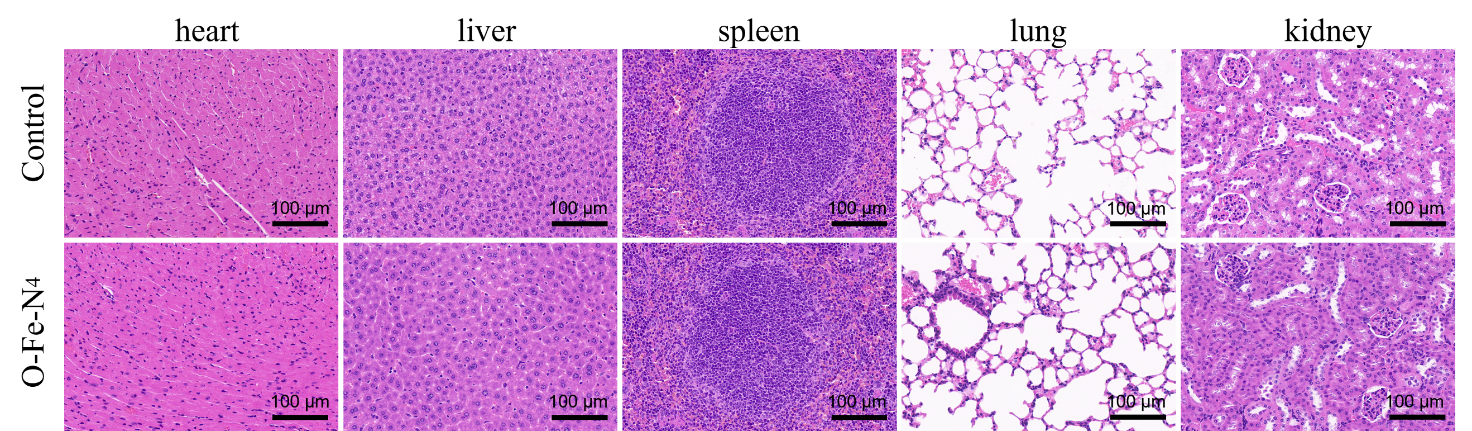


**Figure S40.** Histological sections of the heart, liver, spleen, lung, and kidney from the mice sacrificed 30 days postinjection of O-Fe-N_4_ (10 mg·kg^−1^) after staining with hematoxylin-eosin.


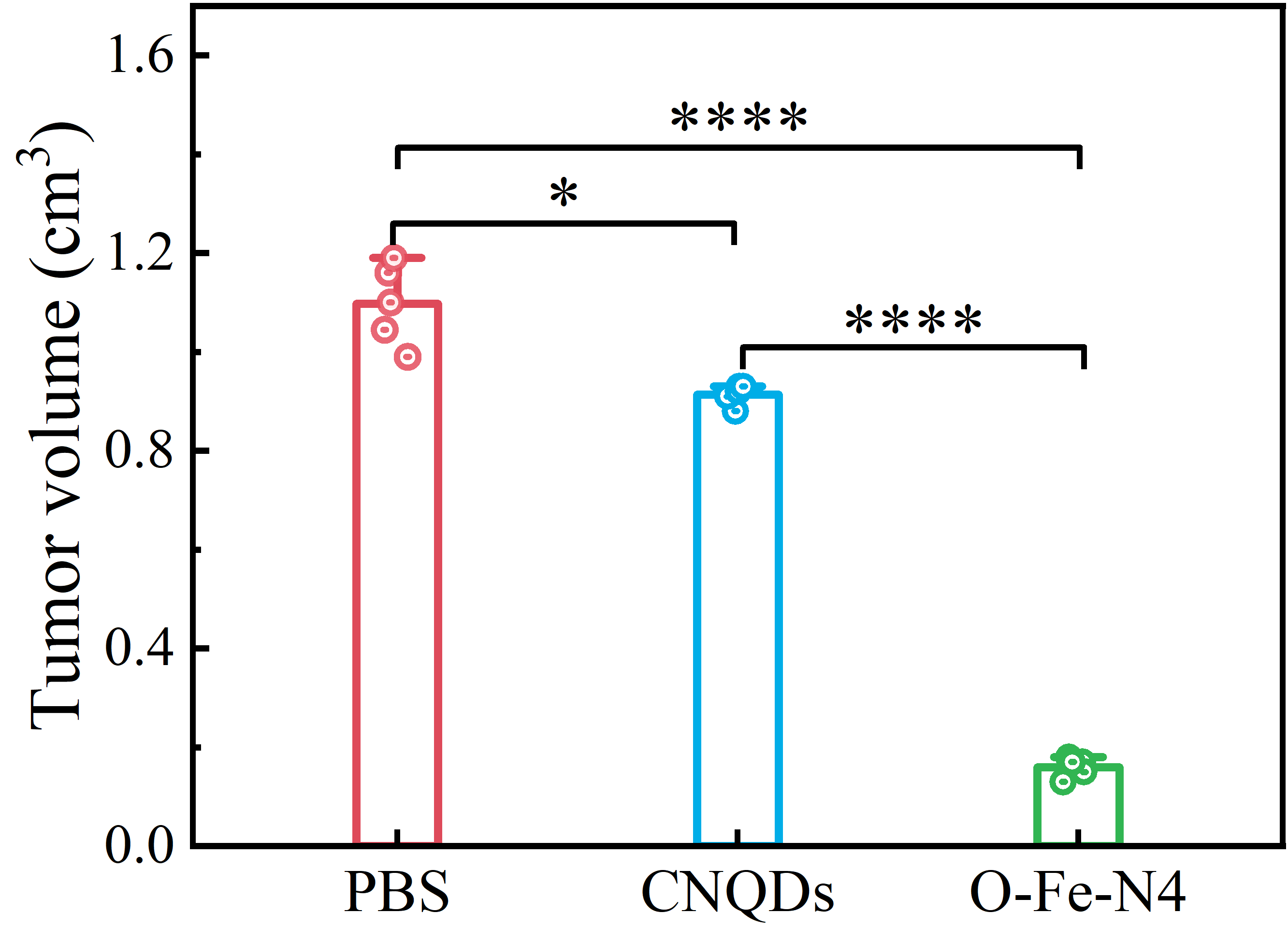


**Figure S41.** The volume of dissected tumors (*n* = 5). Values are expressed as mean ± standard deviation. ****P < 0.0001.

**
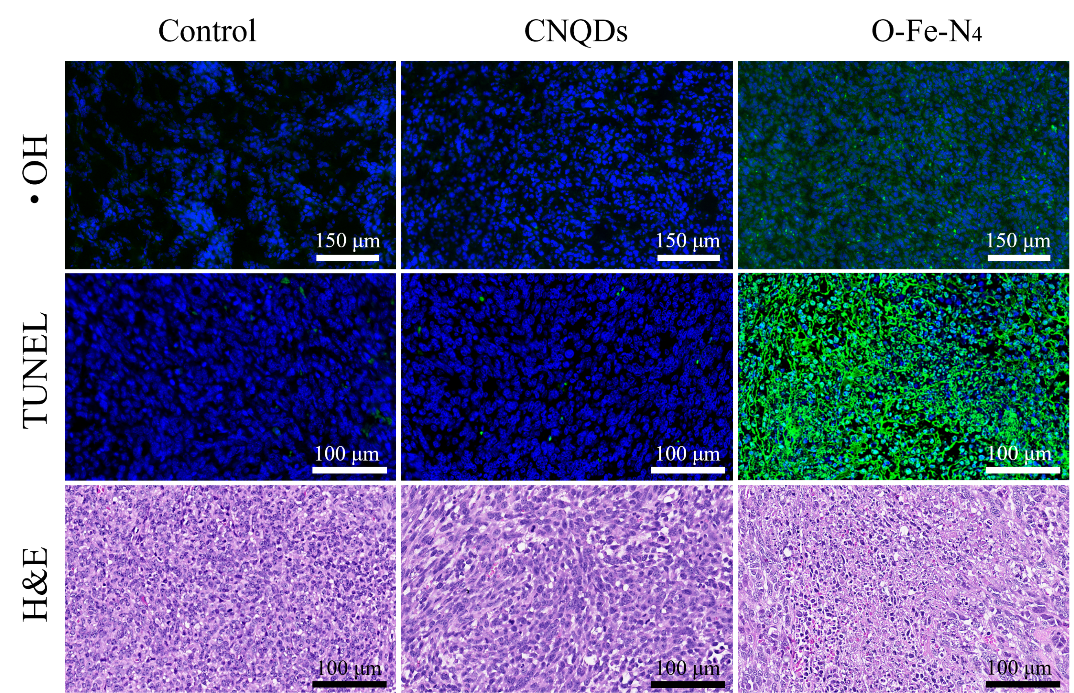
**

**Figure S42.** Immunofluorescence staining of tumors from mice with different treatments.


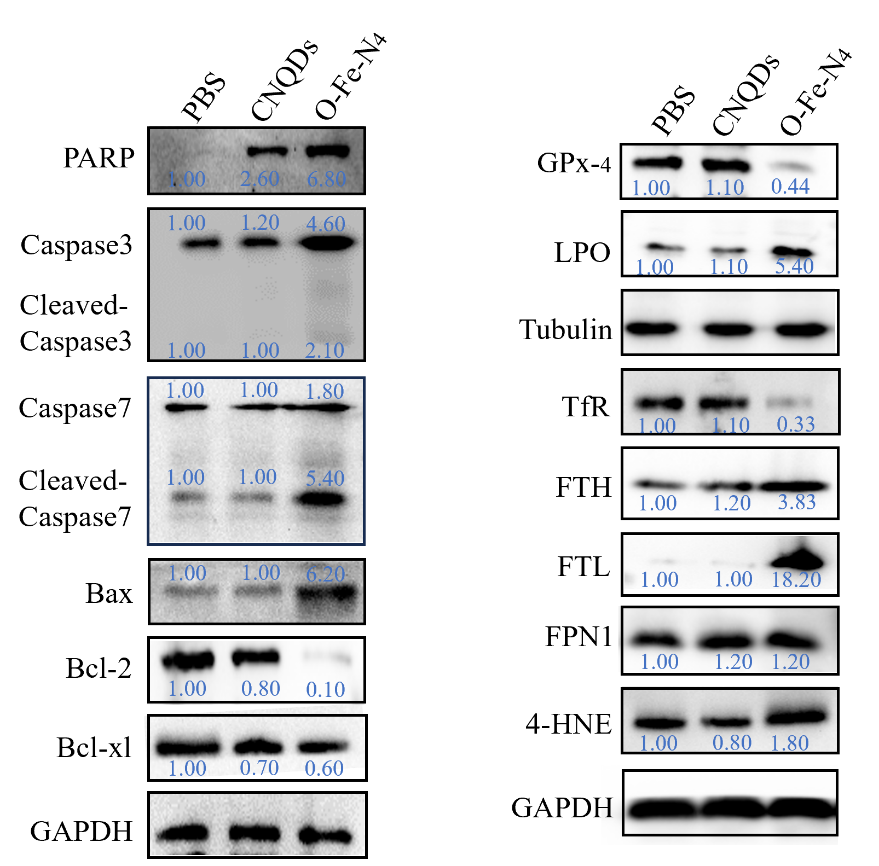


**Figure S43.** Western blotting assay of the representative protein expression of tumors from mice in each group.

1. **Supplementary Table**

**Table S1.** LCF results of O-Fe-N_4_.

| Sample | Weight of standards | | Valance | R factor |
| --- | --- | --- | --- | --- |
| O-Fe-N_4_ | FeO (0.21 ± 0.01) | Fe_2_O_3_ (0.79 ± 0.01) | +2.8 | 0.002 |

**Table S2.** EXAFS fitting parameters at the Fe K-edge (*Ѕ*_0_^2^=0.87, 0.68)

| Sample | Path | C.N. | R (Å) | σ^2^×10^3^ (Å^2^) | ΔE (eV) | R factor |
| --- | --- | --- | --- | --- | --- | --- |
| Fe foil | Fe-Fe | 8* | 2.47 | 4.5 | -2.4 | 0.002 |
|  | Fe-Fe | 6* | 2.84 | 5.2 | -4.6 |  |
| Fe_2_O_3_ | Fe-O | 6.4 | 1.96 | 12.9 | -7.4 | 0.009 |
|  | Fe-Fe | 6.3 | 3.00 | 8.8 | 3.6 |  |
|  | Fe-Fe | 2.6 | 3.67 | 2.3 | -7.9 |  |
| FePc | Fe-N | 4.4 | 1.95 | 5.1 | 0.3 | 0.007 |
|  | Fe-C | 2.2 | 2.95 | 4.6 | 4.4 |  |
| O-Fe-N_4_ | Fe-N | 4* | 1.98±0.05 | 8.3±8.3 | -2.0±2.7 | 0.013 |
|  | Fe-O | 1* | 2.01±0.05 | 13.3±17.4 |  |  |

*C.N.*: coordination numbers; *R*: bond distance; *σ*^2^: Debye-Waller factors; Δ*E*: the inner

potential correction. *R* factor: goodness of fit. * fitting with fixed parameters.

**Table S3.** Comparison of SA and Vmax (H_2_O_2_) of reported SACs nanozymes

| Moiety | Fe (wt%) | pH | SA (U mg^-1^) | reference |
| --- | --- | --- | --- | --- |
| Fe-N_4_ | 6.36 | 4.0 | 4.64 | ^[1]^ |
| Fe-SAEs | 1.20 | 3.8 | 6.75 | ^[2]^ |
| B-doped Fe-N_4_ | 0.6 | 3.0 | 15.41 | ^[3]^ |
| Fe-N_4_ | 13.5 | 3.0 | 25.33 | ^[4]^ |
| - | 5.85 | 3.6 | 36.60 | ^[5]^ |
| Fe-based MOFs | - | 3.0 | 37.50 | ^[6]^ |
| Fe-N_4_ | 8.75 | 6.49 | 41.71 | ^[7]^ |
| Fe-N_4_ | 2.05 | 3.0 | 42.80 | ^[8]^ |
| Fe-N_4_ | 2.12 | 3.6 | 48.5 | ^[9]^ |
| Fe-N_4_ | 1.85 | 3.6 | 57.76 | ^[10]^ |
| FeN_4_ -axial ligand | 5.00 | 3.5 | 67.3 | ^[11]^ |
| Fe-N_3_S_1_ | 1.30 | 3.5 | 79.71 | ^[12]^ |
| P-doped Fe-N_4_ | 3.60 | 3.6 | 86.9 | ^[13]^ |
| FeN_4_C-SO*x* | 0.99 | 3.6 | 119.77 | ^[14]^ |
| FeN_3_P | 2.59 | 3.6 | 316 | ^[15]^ |
| O-Fe-N_4_ | 5.26 | 6.5 | 79.58 | This work |

**Note**:

1. The SA value of P-doped Fe-N_4_ at pH 6.5 is approximately ~87% of f that value at pH 3.6.
2. The SA value of FeN_4_C-So*x* at pH 6.5 is approximately 30%-40% of that value at pH 3.6.

**References:**

[1] C. Zhang, C. Chen, D. Zhao, G. Kang, F. Liu, F. Yang, Y. Lu, J. Sun, *Anal. Chem.* **2022**, 94, 3485.

[2] C. Zhao, C. Xiong, X. Liu, M. Qiao, Z. Li, T. Yuan, J. Wang, Y. Qu, X. Wang, F. Zhou, Q. Xu, S. Wang, M. Chen, W. Wang, Y. Li, T. Yao, Y. Wu, Y. Li, *Chem. Commun.* **2019**, 55, 2285.

[3] L. Jiao, W. Xu, Y. Zhang, Y. Wu, W. Gu, X. Ge, B. Chen, C. Zhu, S. Guo, *Nano Today* **2020**, 35, 100971.

[4] L. Jiao, J. Wu, H. Zhong, Y. Zhang, W. Xu, Y. Wu, Y. Chen, H. Yan, Q. Zhang, W. Gu, L. Gu, S. P. Beckman, L. Huang, C. Zhu, *ACS Catal.* **2020**, 10, 6422.

[5] J. Hao, C. Zhang, C. Feng, Q. Wang, Z.-Y. Liu, Y. Li, J. Mu, E.-C. Yang, Y. Wang, *Chin. Chem. Lett.* **2023**, 34, 107650.

[6] W. Xu, Y. Kang, L. Jiao, Y. Wu, H. Yan, J. Li, W. Gu, W. Song, C. Zhu, *Nanomicro Lett.* **2020**, 12, 184.

[7] X. Zhu, J. Wu, R. Liu, H. Xiang, W. Zhang, Q. Chang, S. Wang, R. Jiang, F. Zhao, Q. Li, L. Huang, L. Yan, Y. Zhao, *ACS Nano* **2022**, 16, 18849.

[8] S. Ding, Z. Lyu, L. Fang, T. Li, W. Zhu, S. Li, X. Li, J. C. Li, D. Du, Y. Lin, *Small* **2021**, 17, e2100664.

[9] Z. Lyu, S. Ding, M. Wang, X. Pan, Z. Feng, H. Tian, C. Zhu, D. Du, Y. Lin, *Nanomicro Lett.* **2021**, 13, 146.

[10] X. Niu, Q. Shi, W. Zhu, D. Liu, H. Tian, S. Fu, N. Cheng, S. Li, J. N. Smith, D. Du, Y. Lin, *Biosens. Bioelectron.* **2019**, 142, 111495.

[11] W. Xu, W. Song, Y. Kang, L. Jiao, Y. Wu, Y. Chen, X. Cai, L. Zheng, W. Gu, C. Zhu, *Anal. Chem.* **2021**, 93, 12758.

[12] L. Jiao, Y. Kang, Y. Chen, N. Wu, Y. Wu, W. Xu, X. Wei, H. Wang, W. Gu, L. Zheng, W. Song, C. Zhu, *Nano Today* **2021**, 40, 101261.

[13] S. Ding, J. A. Barr, Z. Lyu, F. Zhang, M. Wang, P. Tieu, X. Li, M. H. Engelhard, Z. Feng, S. P. Beckman, X. Pan, J. C. Li, D. Du, Y. Lin, *Adv. Mater.* **2023**, e2209633.

[14] G. Li, H. Liu, T. Hu, F. Pu, J. Ren, X. Qu, *J. Am. Chem. Soc.* **2023**, 145, 16835.

[15] S. Ji, B. Jiang, H. Hao, Y. Chen, J. Dong, Y. Mao, Z. Zhang, R. Gao, W. Chen, R. Zhang, Q. Liang, H. Li, S. Liu, Y. Wang, Q. Zhang, L. Gu, D. Duan, M. Liang, D. Wang, X. Yan, Y. Li, *Nat. Catal.* **2021**, 4, 407.
